# Drug Discovery of Nucleos(t)ide Antiviral Agents: Dedicated to Prof. Dr. Erik De Clercq on Occasion of His 80th Birthday

**DOI:** 10.3390/molecules26040923

**Published:** 2021-02-09

**Authors:** Guangdi Li, Tingting Yue, Pan Zhang, Weijie Gu, Ling-Jie Gao, Li Tan

**Affiliations:** 1 Department of Laboratory Medicine, The Second Xiangya Hospital, Central South University, Changsha 410011, China; liguangdi.research@gmail.com; 2 Hunan Provincial Key Laboratory of Clinical Epidemiology, Xiangya School of Public Health, Central South University, Changsha 410078, China; yuetingting@csu.edu.cn (T.Y.); paulazhang@csu.edu.cn (P.Z.); 3 Laboratory of Medicinal Chemistry, Rega Institute for Medicinal Research, KU Leuven, 3000 Leuven, Belgium; weijie.gu@kuleuven.be (W.G.); Ling-jie.gao@kuleuven.be (L.-J.G.)

**Keywords:** antiviral therapy, nucleoside analogue, nucleotide analogue, HIV, HBV, HCV, HSV, VZV, HCMV

## Abstract

Nucleoside and nucleotide analogues are essential antivirals in the treatment of infectious diseases such as human immunodeficiency virus (HIV), hepatitis B virus (HBV), hepatitis C virus (HCV), herpes simplex virus (HSV), varicella-zoster virus (VZV), and human cytomegalovirus (HCMV). To celebrate the 80th birthday of Prof. Dr. Erik De Clercq on 28 March 2021, this review provides an overview of his contributions to eight approved nucleos(t)ide drugs: (i) three adenosine nucleotide analogues, namely tenofovir disoproxil fumarate (Viread^®^) and tenofovir alafenamide (Vemlidy^®^) against HIV and HBV infections and adefovir dipivoxil (Hepsera^®^) against HBV infections; (ii) two thymidine nucleoside analogues, namely brivudine (Zostex^®^) against HSV-1 and VZV infections and stavudine (Zerit^®^) against HIV infections; (iii) two guanosine analogues, namely valacyclovir (Valtrex^®^, Zelitrex^®^) against HSV and VZV and rabacfosadine (Tanovea^®^-CA1) for the treatment of lymphoma in dogs; and (iv) one cytidine nucleotide analogue, namely cidofovir (Vistide^®^) for the treatment of HCMV retinitis in AIDS patients. Although adefovir dipivoxil, stavudine, and cidofovir are virtually discontinued for clinical use, tenofovir disoproxil fumarate and tenofovir alafenamide remain the most important antivirals against HIV and HBV infections worldwide. Overall, the broad-spectrum antiviral potential of nucleos(t)ide analogues supports their development to treat or prevent current and emerging infectious diseases worldwide.

## 1. Introduction

**Figure 1:**
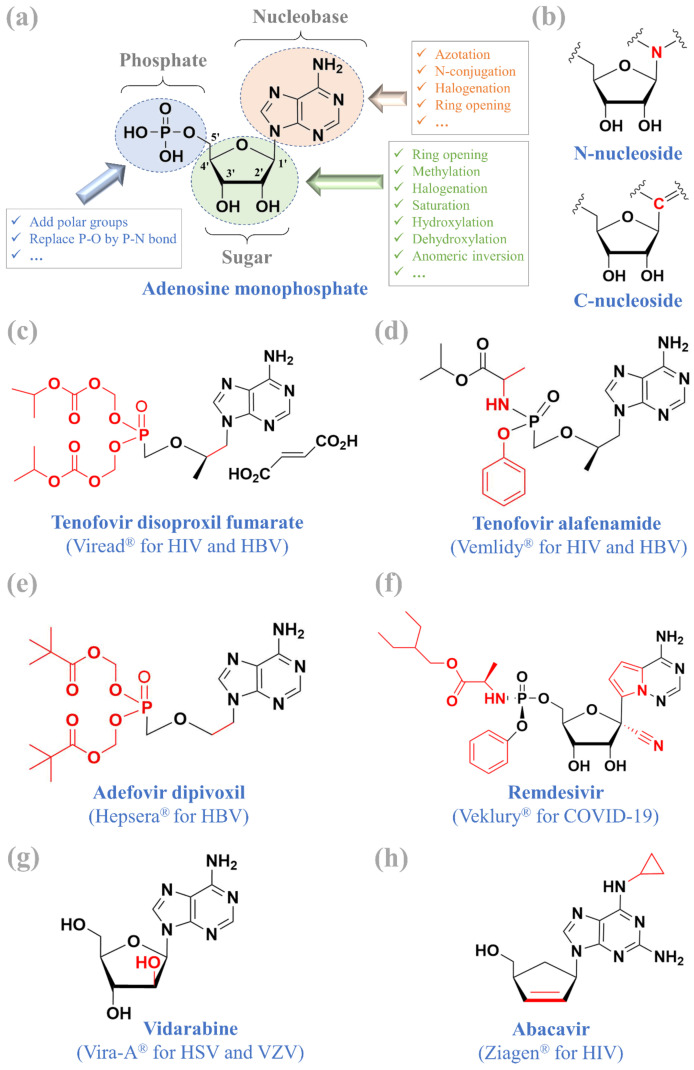
Adenosine monophosphate (**a**), N-nucleoside and C-nucleoside (**b**), and six approved adenosine nucleos(t)ide analogues (**c**–**h**).

Nucleoside and nucleotide analogues are important antiviral, antiparasitic, and anticancer therapeutics that have been widely applied in clinical practice [1,2,3,4]. As shown in Figure 1a, nucleosides are formed by a sugar moiety and a nucleobase, while nucleotides are composed of a sugar moiety, a nucleobase, and at least one phosphate or phosphate-like group [4]. Nucleoside and nucleotide analogues with modified changes can mimic the structure of nature nucleosides and nucleotides that can be recognized by cellular or viral enzymes [5,6]. Many strategies (e.g., ring-opening, *N*-conjugation, halogenation) have been proposed to design novel nucleoside or nucleotide analogues, such as the acyclic fleximer analogues [7], propargylated purine deoxynucleosides [8], 4′-thionucleosides [9], 3′-fluoro-5′-norcarbocyclic nucleoside phosphonates [10], 4′-modified-2′-deoxy-2′-fluoro nucleosides [11], uracil-containing heterodimers [12], l-dideoxy bicyclic pyrimidine nucleoside analogues [13], and imidazo[4,5-b]pyridine nucleoside analogues [14]. In addition to the development of *N*-nucleoside analogues, C-nucleoside analogues could be considered because they are stably resistant against the phosphorolytic degradation caused by phosphorylases [15]. Of note, a *N*-nucleoside links its sugar moiety to its nucleobase through a nitrogen, while a C-nucleoside uses a carbon atom that substitutes the nitrogen to link the sugar moiety and the nucleobase (Figure 1b).

As of today, Prof. Dr. Erik De Clercq has contributed to the approval of nine small-molecule compounds that can be applied to treat viral infections and cancers such as multiple myeloma and non-Hodgkin’s lymphoma (Table 1). Based on their chemical structures, these nine approved drugs can be arbitrarily classified into adenosine nucleotide analogues (tenofovir disoproxil fumarate, tenofovir alafenamide, adefovir dipivoxil), thymidine nucleoside analogues (stavudine, brivudine), guanosine analogues (valacyclovir, rabacfosadine), one cytidine nucleotide analogue (cidofovir), and one bicyclam derivative (plerixafor). Regarding their mechanisms of antiviral action, nucleoside and nucleotide analogues mimic the structure of a natural nucleoside that can be recognized by cellular and viral enzymes such as DNA and RNA polymerases, and due to their structural modifications, they also disrupt or terminate viral replications or biological processes [3,5]. Additionally, the CXCR4 inhibitor plerixafor blocks the interaction of CXCR4 with its natural ligand CXCL12, thereby mobilizing the CD34+ stem cells from the bone marrow into the peripheral bloodstream [16,17]. Because plerixafor was previously reviewed by our team and our beloved professor [16,17,18,19,20,21], this review will focus on nucleos(t)ide antiviral agents in four categories: (i) adenosine nucleos(t)ide analogues, (ii) thymidine nucleos(t)ide analogues, (iii) guanosine nucleos(t)ide analogues, and (iv) cytidine nucleos(t)ide analogues.

To celebrate the 80th anniversary of our beloved professor Erik De Clercq on 28 March 2021, this review provides an overview of nucleoside and nucleotide analogues. We searched his publications between 1967 and 2020. The reference collection of research articles and related books was extracted from databases such as PubMed, Science Direct, and Google Scholar. The drug information was extracted from the US FDA website. Research websites (www.virusface.com, www.erikdeclercq.org) were used to update the teaching lectures and publications of our beloved professor Erik De Clercq.

## 2. Adenosine Nucleos(t)ide Analogues

As of February 2021, at least six adenosine nucleos(t)ide analogues have been officially approved for clinical use, including (i) tenofovir disoproxil fumarate (Viread^®^) for HIV and HBV treatment, (ii) tenofovir alafenamide (Vemlidy^®^) for HIV and HBV treatment, (iii) adefovir dipivoxil (Hepsera^®^) for HBV treatment, (vi) abacavir (Ziagen^®^) for HIV treatment, (v) vidarabine (Vira-A^®^, which is now discontinued) for HSV and VZV treatment, and (vi) remdesivir (Veklury^®^) for COVID-19 treatment (Figure 1). This section focuses on the former three adenosine analogues contributed by Prof. Erik De Clercq, while the other drugs have been reviewed in previous studies [3,22,23,24,25].

### 2.1. Tenofovir Disoproxil Fumarate and Tenofovir Alafenamide

Tenofovir disoproxil fumarate (Figure 1b) and tenofovir alafenamide (Figure 1c) are the most popular antivirals in the treatment of HIV and HBV infections worldwide. In 1993, the antiviral activity of tenofovir ((R)-9-(2-phosphonylmethoxypropyl)adenine, (R)-PMPA) was described to inhibit both HIV-1 (EC_50_ = 5.9 ± 0.45 μM) and HIV-2 (EC_50_ = 4.9 ± 0.45 μM) in MT-4 cell cultures [26]. In rhesus macaques, the subcutaneous injection of tenofovir either 48 h before, 4 h after, or 24 h after virus inoculation prevented the infection of simian immunodeficiency virus without toxicity [27]. To increase their oral bioavailability, tenofovir was later derivatized to its oral prodrug form called tenofovir disoproxil (Figure 1b). Tenofovir disoproxil undertakes two intracellular phosphorylations to become the active metabolite of tenofovir diphosphates that act as chain terminators to block HIV reverse transcriptase and HBV DNA polymerase [28]. Since its approval in 2001, tenofovir disoproxil fumarate has been widely applied as the backbone of highly active antiretroviral therapy (HAART) [29] in HIV treatments, including the following:

- Viread^®^ (tenofovir disoproxil fumarate);
- Truvada^®^ (tenofovir disoproxil fumarate, emtricitabine);
- Atripla^®^ (tenofovir disoproxil fumarate, emtricitabine, efavirenz);
- Eviplera^®^ (tenofovir disoproxil fumarate, emtricitabine, rilpivirine hydrochloride);
- Stribild^®^ (tenofovir disoproxil fumarate, emtricitabine, elvitegravir, cobicistat);
- Symfi^®^ (tenofovir disoproxil fumarate, lamivudine, efavirenz 600 mg);
- Symfi Lo^®^ (tenofovir disoproxil fumarate, lamivudine, efavirenz 400 mg);
- Cimduo^®^ (tenofovir disoproxil fumarate, lamivudine);
- Delstrigo^®^ (tenofovir disoproxil fumarate, lamivudine, doravirine).

Tenofovir alafenamide is a promising successor of tenofovir disoproxil fumarate [30]. With a lower dose, tenofovir alafenamide 25 mg induces a lower risk of kidney toxicity and bone density changes and offers long-term potential in the HIV preexposure prophylaxis [28]. In HIV-infected patients, tenofovir alafenamide 25 mg is superior to tenofovir disoproxil fumarate 300 mg regarding the efficacy and safety profiles [31]. As the replacement of tenofovir disoproxil fumarate, tenofovir alafenamide has been applied as the backbone of HAART in current HIV treatments, including the following:

- Vemlidy^®^ (tenofovir alafenamide);
- Descovy^®^ (tenofovir alafenamide, emtricitabine);
- Genvoya^®^ (tenofovir alafenamide, emtricitabine, elvitegravir, cobicistat);
- Odefsey^®^ (tenofovir alafenamide, emtricitabine, rilpivirine hydrochloride);
- Biktarvy^®^ (tenofovir alafenamide, emtricitabine, bictegravir);
- Symtuza^®^ (tenofovir alafenamide, emtricitabine, darunavir, cobicistat).

Truvada was approved by the US FDA in 2012 for the prevention of HIV infection, making it the first approved regimen for HIV prevention. In the iPrEx trial that enrolled HIV-seronegative men or transgender women who had sex with men, a 44% reduction of HIV incidences was observed in the Truvada group compared with that in the placebo group (36/1251 versus 64/1248) [32]. Similar to tenofovir disoproxil fumarate plus emtricitabine (Truvada^®^), tenofovir alafenamide plus emtricitabine (Descovy^®^) are also approved for HIV-1 prophylaxis by the US FDA.

In the treatment of HBV infections, both tenofovir disoproxil fumarate and tenofovir alafenamide are popular antiviral drugs in clinical practice. Compared with the approved compound entecavir, tenofovir disoproxil fumarate offered more frequent elastographic reversion of cirrhosis at year 5 [33]. In randomized trials of HBeAg-positive and HBeAg-negative patients, tenofovir alafenamide 25 mg improved renal and bone safety compared with tenofovir disoproxil fumarate 300 mg [34,35]. Moreover, drug resistance mutations were not observed after a 2-year treatment of tenofovir alafenamide 25 mg [36]. The most common adverse events of tenofovir disoproxil fumarate include laboratory abnormalities (19%), gastrointestinal disorders (15%), infestations and infections (13%), and nervous system disorders (8%) [37]. Similarly, the most frequent adverse events of tenofovir alafenamide are gastrointestinal disorders (23%), laboratory abnormalities (20%), infestations and infections (18%), renal and urinary disorders (11%), and nervous system disorders (9%) [37]. Compared with tenofovir alafenamide, tenofovir disoproxil fumarate caused a higher risk of bone loss in the hip and spine [37].

### 2.2. Adefovir Dipivoxil

In 2002, adefovir dipivoxil 10 mg (Hepsera^®^) once daily orally with or without food was approved for HBV infections. Adefovir dipivoxil was initially designed to inhibit HIV, but its HIV application was abandoned because of its nephrotoxicity and its inferiority to tenofovir disoproxil fumarate [38]. Adefovir dipivoxil was subsequently pursued for HBV treatment, and its dosage of 10 mg/day was effective to treat HBeAg-positive and HBeAg-negative patients [39,40]. In randomized clinical trials, 48 weeks of adefovir dipivoxil 10 mg/day offered significant virologic, histologic, and biochemical improvement, while adefovir-associated resistance mutations were not identified in the HBV DNA polymerase [39,40]. However, a daily dose of adefovir dipivoxil 10 mg was inferior to a daily dose of tenofovir disoproxil fumarate 300 mg through 48 weeks [41]. Five-year treatment of tenofovir disoproxil fumarate also offered better safety and efficacy in HBV-infected patients [42]. According to the AASLD and EASL guidelines of HBV management, adefovir dipivoxil has been virtually replaced by tenofovir disoproxil fumarate and tenofovir alafenamide due to their better clinical efficacy and safety profiles [43,44].

### 2.3. Novel Adenosine Nucleos(t)ide Analogues

In addition to the approved drugs above, many adenosine nucleos(t)ide analogues are still under development. For instance, (i) a new nucleoside derivate called E-CFCP ((1S,3S,5S,E)-3-(2-amino-6-oxo-1,6-dihydro-9*H*-purin-9-yl)-2-(fluoromethylene)-5-hydroxy-1-(hydroxymethyl) cyclopentane-1-carbonitrile) was recently proposed to offer long-acting activities against HBV [45]. With its strong penetration ability to hepatocytes, E-CFCP showed its once-weekly dosing potential and exhibited less toxicity than tenofovir alafenamide and entecavir [45]. Due to its exquisite electronegativity, fluorine could significantly change the steric and electronic features of the nucleoside sugar ring and increase the stability of neighboring bounds [46]. (ii) 3′-Deoxy-3′-fluoroadenosine is a fluorine-substituted analogue with low-micromolar antiviral activities against tick-borne encephalitis virus and West Nile virus (EC_50_ = 1.6 to 4.7 μM) [46]. This compound also significantly reduced mortality and eliminated neuroinfections in a mouse model infected with West Nile virus [46]. (iii) The bicyclo[2.2.1]heptane skeleton was recently proposed for the sugar moiety in nucleoside analogues [47]. Despite their high cytotoxicity, the most promising analogues (6f, 6d, and 6j) showed similar levels of IC_50_ to that of acyclovir against HSV-1 and enterovirus 71 [47].

## 3. Thymidine Nucleos(t)ide Analogues

**Figure 2:**
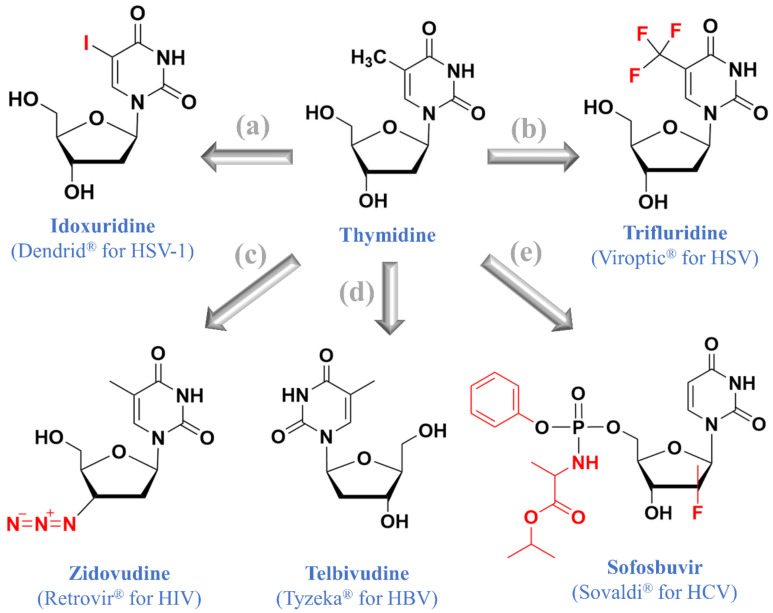
Thymidine and five approved thymidine nucleos(t)ide analogues: (**a**) idoxuridine, (**b**) trifluridine, (**c**) zidovudine, (**d**) telbivudine, and (**e**) sofosbuvir.

**Figure 3:**
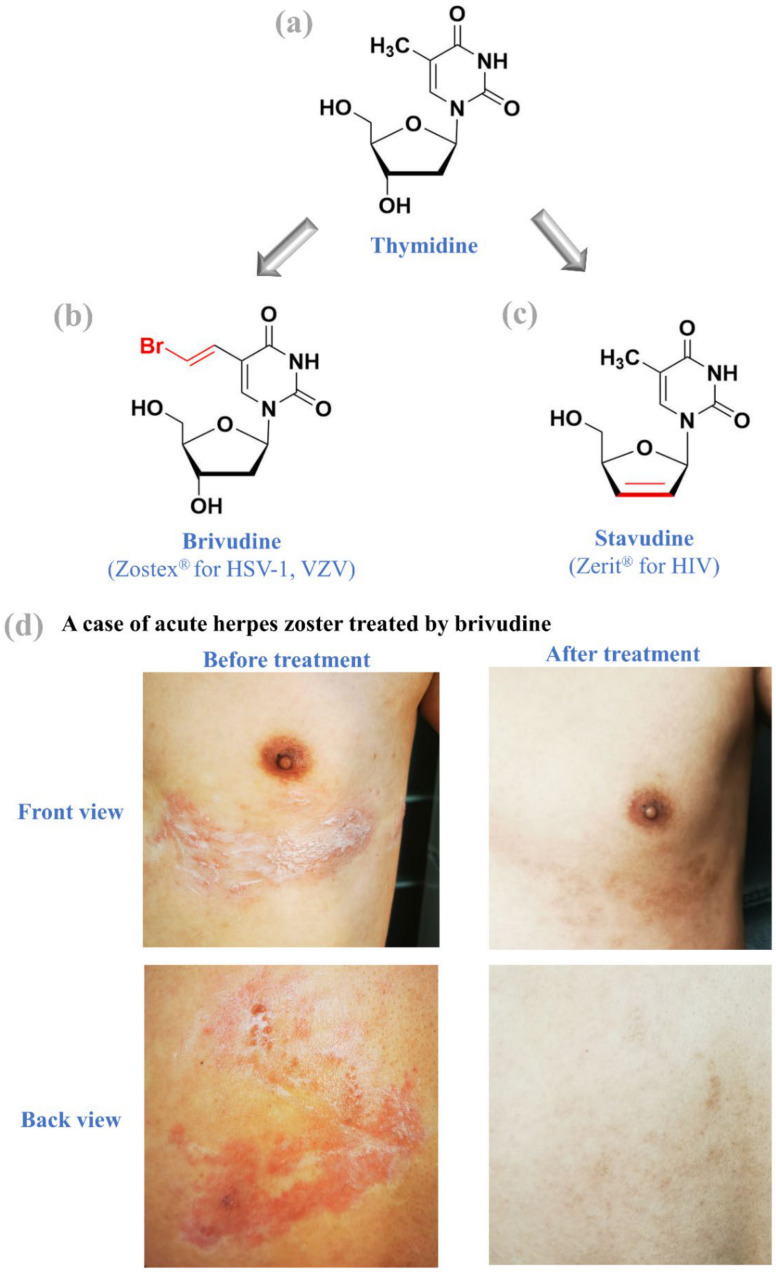
Thymidine and thymidine nucleoside analogues. Development from thymidine (**a**) to brivudine (**b**) and stavudine (**c**). A case of acute herpes zoster was treated by brivudine (**d**). In the first 3 days after symptom onset, a corticosteroid topical cream (mometasone furoate) offered no improvement. Based on the advice of our beloved professor Erik De Clercq, three once-daily tablets of brivudine (Zostex^®^) were subsequently used to cure the herpes zoster, and all crusts fell off 3 weeks later. Front and back views were taken before the brivudine treatment and 2 months after the treatment.

As of today, there are at least seven thymidine nucleos(t)ide analogues approved for clinical use, including (i) brivudine (Zostex^®^) for HSV-1 and VZV treatment, (ii) stavudine (Zerit^®^) for HIV treatment, (iii) idoxuridine (Dendrid^®^) for HSV-1 treatment, (iv) trifluridine (Viroptic^®^) for HSV treatment, (v) zidovudine (Retrovir^®^) for HIV treatment, (vi) telbivudine (Tyzeka^®^) for HBV treatment, and (vii) sofosbuvir (Sovaldi^®^) for HCV treatment (Figure 2 and Figure 3). As described by previous reviews [48,49], the career of Prof. Dr. Erik De Clercq began with interferon inducers and then shifted to nucleos(t)ide analogues such as stavudine and brivudine (Figure 3). Here, we focus on brivudine and stavudine, while other compounds have been reviewed in our previous study [3].

### 3.1. Brivudine

While brivudine ((E)-5-(2-bromovinyl)-2′-deoxyuridine) was discovered in 1976 at the University of Birmingham, its antiviral activities in cell cultures and clinical reports were first reported in 1979 by Erik De Clercq et al. [50]. After its phosphorylation by the viral thymidine kinase and nucleoside-diphosphate kinase, the brivudine 5′-triphosphate blocks the incorporation of viral DNA and inhibits the activity of viral DNA polymerases [3]. In both cell cultures and animal models, brivudine exerted a remarkable inhibitory effect on the replication of HSV-1 and VZV [50,51]. Moreover, the anti-VZV activity of brivudine was much more potent than that of (val)acyclovir, ganciclovir, and penciclovir [49,52].

After its rocky journey of clinical development [48], brivudine was approved in many countries (e.g., Germany, Belgium, Czech Republic, and Greece) to treat herpes zoster caused by VZV and HSV-1 (note that HSV-2 often causes genital herpes). In clinical practice, the once-daily tablets of brivudine should be administered as early as possible, preferably within 72 h from the first cutaneous manifestations such as red blistery patches. According to the label instructions, the safety and efficacy of brivudine in a 7-day course of therapy have been validated in adults, but its clinical effectiveness in the pediatric population (age 0 to 18 years) remains unclear. Figure 3d shows a case of herpes zoster treated by brivudine—as experienced firsthand by our team member who received the antiviral drugs contributed by Prof. Erik De Clercq.

### 3.2. Stavudine

Shortly after the anti-HIV discovery of zidovudine (AZT) in October 1985, stavudine (d4T) was discovered simultaneously at three locations: the Rega Institute, Yale University, and Yamamoto’s Laboratory in Tokyo [49]. However, the anti-HIV activity of stavudine (2′,3′-didehydro-2′,3′-dideoxythymidine) was first described in 1987 by Masanori Baba et al. [53], who was, at that time, a favorite student of Prof. Erik De Clercq. Stavudine lacks the 3′-hydroxyl group (Figure 3c) which is indispensable for chain elongation. The incorporation of stavudine into nascent viral DNA causes the termination of the HIV transcription [54]. In 1994, stavudine was approved by the US FDA. However, a high level of drug resistance can be induced through the increased phosphorolytic excision of the incorporated monophosphate of stavudine [54]. Due to its off-target toxicity levels and drug resistance, stavudine was discontinued and removed from the market in 2020.

## 4. Guanosine Nucleos(t)ide Analogues

**Figure 4:**
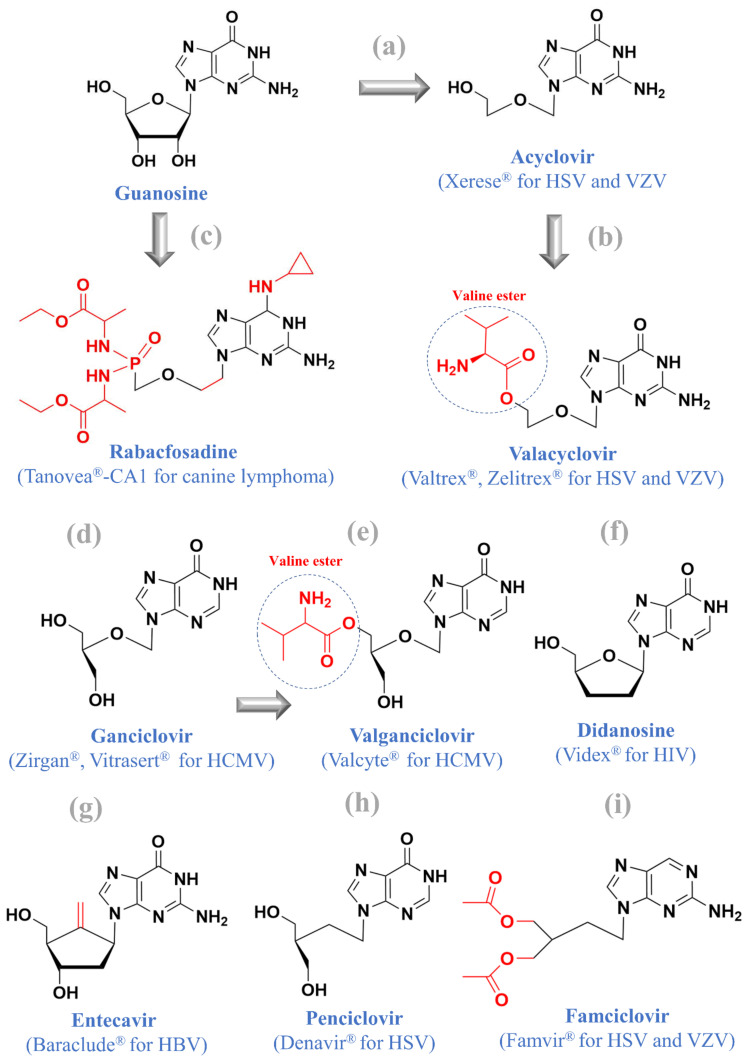
Guanosine and its nine approved analogues: (**a**) acyclovir, (**b**) valacyclovir, (**c**) rabacfosadine, (**d**) ganciclovir, (**e**) valganciclovir, (**f**) didanosine, (**g**) entecavir, (**h**) penciclovir, and (**i**) famciclovir.

Currently, there are at least eight approved guanosine nucleos(t)ide analogues, including (i) valacyclovir (Valtrex^®^) for HSV and VZV treatment, (ii) rabacfosadine (Tanovea^®^-CA1) for treating lymphoma in dogs, (iii) didanosine (Videx^®^) for HIV treatment, (iv) entecavir (Baraclude^®^) for HBV treatment, (v) famciclovir (Famvir^®^) for HSV and VZV treatment, (vi) penciclovir (Denavir^®^) for HSV treatment, (vii) ganciclovir (Zirgan^®^, Vitrasert^®^) for HCMV treatment, and (viii) valganciclovir (Valcyte^®^) for HCMV treatment (Figure 4). Novel compounds such as l-dideoxy bicyclic pyrimidine nucleoside analogues for Zika virus infections have also been developed [13]. Here, we focus on valacyclovir and rabacfosadine, while other compounds have been reviewed in our previous study [3].

### 4.1. Valacyclovir

Valacyclovir (or valaciclovir) is the derivative of acyclovir with the valine ester (Figure 4a,b), which was co-discovered in 1983 by Leon Colla, Erik De Clercq, Roger Busson, and Hubert Vanderhaeghe at the Rega Institute [55]. This strategy was later applied to design valganciclovir by adding the valine ester to ganciclovir (Figure 4d,e). Before the advent of valacyclovir, acyclovir was the gold standard in the 1980s and was widely applied in the treatment of herpesvirus infections [56]. At the very beginning, Prof. Erik De Clercq thought the modifications of an existing compound to its prodrug were not very innovative [56], but valacyclovir with the aminoacyl ester surprisingly showed better oral absorption than acyclovir because of its increased aqueous solubility (51% to 54%) compared to the parent compound [55,57]. This also supports the use of oral applications of valacyclovir to treat cold sores (2 g every 12 h for 1 day) over acyclovir (5 times per day for 5 days).

In 1995, valacyclovir was approved by the US FDA for the treatment of cold sores, herpes zoster, and chickenpox. Compared with the oral acyclovir, valacyclovir enhances the bioavailability of acyclovir (10% to 20% according to dose), while acyclovir and valacyclovir share comparable safety profiles in the treatment of HSV infections [58]. Moreover, valacyclovir may play a promising role in the antenatal treatment of congenital cytomegalovirus [59] as well as the antiviral prophylaxis to prevent the late onset of HCMV infections in kidney–pancreas EBV-seronegative kidney recipients [60].

### 4.2. Rabacfosadine

In 2016, rabacfosadine (GS-9219, VDC-1101) was conditionally approved to treat canine lymphoma using the intravenous injection of 1 mg/kg once every 3 weeks for up to 5 weeks. Rabacfosadine (Figure 4c) is a pro-prodrug of an acyclic guanosine nucleoside phosphonate called PMEG (9-(2-phosphonylmethoxyethyl)guanine). In 1987, the antiviral activity of PMEG was first described by Erik De Clercq et al. [61]. In cell cultures, PMEG acted as an antiviral compound to inhibit HCMV (minimum inhibitory concentration: 0.25 μg/mL), HSV (HSV-1: 4 μg/mL, HSV-2: 7 μg/mL), and VZV (0.05 μg/mL) [61].

Despite its antiviral activity, PMEG was the most cytotoxic compound among phosphonylmethoxyalkyl derivatives, because its diphosphates could be incorporated into host DNA and block cellular DNA polymerase delta and epsilon [62]. A subsequent study revealed the antitumor activity of PMEG and its intracellular prodrug cPr-PMEDAP (9-(2-phosphonylmethoxyethyl)-*N*6-cyclopropyl-2,6-diaminopurine) in a rat choriocarcinoma tumor model [63]. To increase the efficiency of lymphoid cell loading, a phosphonamidate moiety was added to cPr-PMEDAP, leading to the creation of rabacfosadine (diethyl *N*,*N*′-([(2-(2-amino-6-[cyclopropylamino]-9*H*-purin-9-yl)ethoxy)methyl] phosphonoyl) di-L-alaninate) [64]. In five pet dogs with spontaneous, advanced-stage non-Hodgkin’s lymphoma, a single-dose, single-agent, 30-min infusion of rabacfosadine (1 mg/kg) displayed significant in vivo efficacy [65]. Despite its success in treating canine lymphoma, rabacfosadine is currently no longer being pursued for human clinical development.

## 5. Cytidine Nucleos(t)ide Analogues

**Figure 5:**
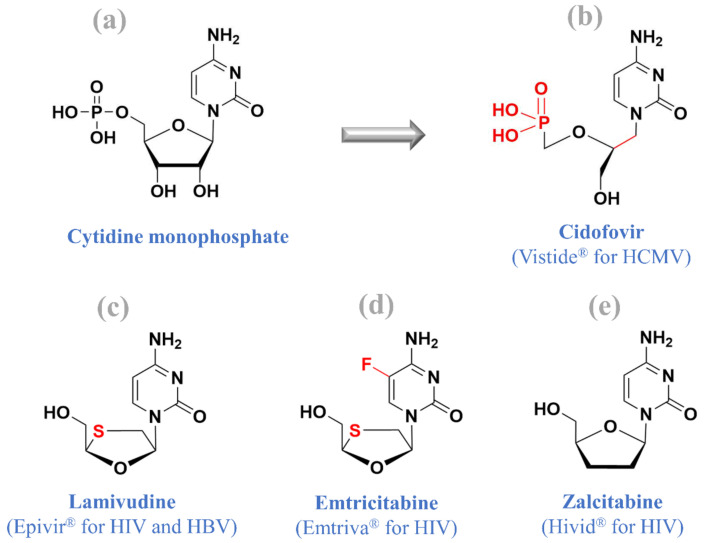
Cytidine monophosphate (**a**) and four approved cytidine nucleos(t)ide analogues: (**b**) cidofovir, (**c**) lamivudine, (**d**) emtricitabine, and (**e**) zalcitabine.

As of February 2021, at least four cytidine nucleos(t)ide analogues have been approved for clinical use, including (i) cidofovir (Vistide^®^) for treating HCMV retinitis in AIDS patients, (ii) zalcitabine (Hivid^®^, which is now discontinued) for HIV treatment, (iii) emtricitabine (Emtriva^®^) for HIV treatment, and (iv) lamivudine (Epivir^®^) for HIV and HBV treatment (Figure 5). This section focuses on cidofovir, a contribution of our beloved professor, while other drugs have been reviewed in our previous article [3].

Cidofovir is an acyclic nucleoside phosphonate (Figure 5b) licensed as an anti-DNA viral agent [66]. In 1987, cidofovir was first reported as an acyclic nucleoside derivative called (S)-HPMPC that effectively inhibited HCMV in human embryonic lung cells (minimum antiviral concentration: 0.08 μg/mL, selectivity index: 625) [61]. In 1996, the intravenous infusion of cidofovir was approved in the treatment of HCMV retinitis in AIDS patients—a severe complication that almost no longer exists thanks to the successful application of HAART treatments [67]. Its off-label use is primarily considered for treating DNA viruses such as Epstein-Barr virus (EBV), poxvirus infections (e.g., molluscum contagiosum), adenovirus infection, and human polyoma infections [67,68]. For instance, two patients with locally recurrent EBV-associated nasopharyngeal carcinoma were successfully treated by cidofovir [69].

## 6. Conclusions

Among the list of eight nucleos(t)ide analogues (Table 1) co-contributed by our beloved professor, adefovir dipivoxil, stavudine, and cidofovir are virtually discontinued for clinical use, whereas tenofovir disoproxil fumarate and tenofovir alafenamide remain the most important antivirals in the treatment of HIV and HBV infections as well as the prophylaxis of HIV infections. By saving millions of HIV-infected or HBV-infected patients worldwide, the popularity of tenofovir disoproxil fumarate and tenofovir alafenamide in clinical practice has proved the potential of nucleotide and nucleoside analogues.

Nucleoside and nucleotide antiviral agents are an important drug class with broad-spectrum antiviral activities to treat current and emerging infectious diseases worldwide, thereby encouraging future development of novel nucleos(t)ide analogues. As a popular example of broad-spectrum antiviral activities, remdesivir inhibits many RNA viruses such as ebolavirus and respiratory pathogens including Middle East respiratory syndrome coronavirus, severe acute respiratory syndrome coronavirus (SARS-CoV), and SARS-CoV-2 [70]. In regards to the worldwide spread of COVID-19 [71,72,73], the US FDA approved remdesivir (Veklury^®^)—a monophosphoramidate prodrug of an adenosine analogue (Figure 1e) that acts as a direct-acting antiviral to treat COVID-19 [74]. Remdesivir, unlike other N-nucleoside analogues from Figure 1, Figure 2, Figure 3, Figure 4 and Figure 5, is a C-nucleoside analogue that is stably resistant against phosphorolytic degradation caused by phosphorylases [15].

In addition to the discovery of broad-spectrum antivirals with better potency, it is important to develop highly selective nucleos(t)ide analogues with minimal toxicity because antiviral nucleos(t)ides may exert toxicity through the disruption of natural nucleoside triphosphate pools and interfere with activities of host polymerases such as human mitochondrial DNA/RNA polymerases (e.g., Pol γ, POLRMT) [75]. Future development of nucleos(t)ide analogues should focus on the reduced toxicity, the increased aqueous solubility, and the reduced risk of drug resistance.

## Figures and Tables

**Table 1:** A list of approved drugs co-invented by Prof. Erik De Clercq.

Drug Name *	Brand Name	Diseases	Clinical Use in Adults	Agency ^‡^	Approval Date
**A** **denosine** **Nucleotide** **Analogues**					
Tenofovir disoproxil fumarate	Viread^®^	HIV, HBV	One 300 mg tablet/day taken orally	U.S. FDA	2001
				EMA	2002
Tenofovir alafenamide	Vemlidy^®^	HIV, HBV	One 25 mg tablet/day taken with food	U.S. FDA	2015
				EMA	2017
Adefovir dipivoxil	Hepsera^®^	HBV	One 10 mg tablet/day taken orally with or without food	U.S. FDA	2002
				EMA	2003
**Thymidine Nucleoside Analogues**					
Stavudine	Zerit^®^	HIV	Adults < 60 kg: 30 mg/per 12 h	U.S. FDA	1994
			Adults ≥ 60 kg: 40 mg/per 12 h	EMA	1996
Brivudine	Zostex^®^ and others	HSV-1, VZV	One 125 mg tablet/day orally for 7 days	‡	2000
**Guanosine Analogues**					
Valacyclovir	Valtrex^®^, Zelitrex^®^	HSV	Cold sores: 2 g/12 h for 1 day	U.S. FDA	1995
			Genital herpes: ≥ 500 mg/day ^#^	EMA	2010
		VZV	1 g three times daily for 7 days		
Rabacfosadine (GS-9219)	Tanovea^®^-CA1	Lymphoma in dogs	1 mg/kg as a 30-min intravenous infusion, once every 3 weeks, for up to five doses	U.S. FDA	2016
**Cytidine Nucleotide Analogue**					
Cidofovir	Vistide^®^	HCMV retinitis (AIDS patients)	Intravenous infusion of 5 mg/kg dose administered biweekly	U.S. FDA	1996
				EMA	1997
**Bicyclam Derivative**					
Plerixafor (AMD3100)	Mozobil^®^	Multiple myeloma, non-Hodgkin’s lymphoma	Initiate Mozobil treatment after G-CSF per day for 4 days. Subcutaneous injection of weight-adjusted Mozobil dose approximately 11 h before the initiation of apheresis up to 4 consecutive days	U.S. FDA	2008
				EMA	2009

* Except rabacfosadine, all the other compounds were approved for treating human diseases. ^‡^ Approval information from the U.S. Food and Drug Administration (FDA) and/or the European Medicines Agency (EMA) was provided with the earliest approval date. Brivudine has been commercialized in many countries under names such as Zostex^®^ (Germany, China, and Turkey), Zostavir^®^ (Czech Republic and Greece), Zerpex^®^ (Belgium), and Brivirac ^®^ (Italy). ^#^ Doses are adjusted according to the disease stages of genital herpes (see the label instructions).
